# Conceptualization and Measurement of Withdrawal Symptoms in Gaming Disorder and Development and Psychometric Validation of the Gaming Withdrawal Symptoms Questionnaire: Cross-Sectional Study

**DOI:** 10.2196/81407

**Published:** 2026-03-31

**Authors:** Sylwia Starzec, Sławomir Śpiewak, Jolanta Starosta, Paweł Strojny

**Affiliations:** 1 Doctoral School in the Social Sciences Jagiellonian University Kraków Poland; 2 Institute of Applied Psychology, Faculty of Management and Social Communication Jagiellonian University Kraków Poland

**Keywords:** behavioral addictions, DSM-5, gaming disorder, IGD-11, internet gaming disorder, psychological assessment, psychological testing, psychometrics, videogame addiction, withdrawal symptoms

## Abstract

**Background:**

Gaming disorder (GD) is an emerging issue that leads to significant impairment, yet existing tools for measuring withdrawal symptoms in GD are limited and often fail to capture its multidimensional nature. Most current measures rely on single-item assessments or adapted tools from substance use disorders, overlooking cognitive, behavioral, and physiological components. A comprehensive, multidimensional questionnaire is needed to more accurately assess withdrawal in GD, aiding in early detection and intervention.

**Objective:**

The objective of this study was to develop and psychometrically validate a comprehensive measurement tool, the Gaming Withdrawal Symptoms Questionnaire (GWSQ), capturing the multidimensional nature of withdrawal symptoms in GD, including affective, cognitive, behavioral, and physiological components.

**Methods:**

A multistage psychometric approach was used, starting with item generation from a scoping literature review. Exploratory factor analysis and confirmatory factor analysis were conducted to refine the questionnaire. Reliability and validity were assessed using 2 cross-sectional studies. Data were collected anonymously via an online survey platform. Participants were recruited from gaming-related platforms and social media (eg, Discord, Reddit, and Facebook) and restricted to actively engaged adult gamers who passed attention check questions to ensure data quality.

**Results:**

Study 1 involved 480 adults (mean age 23, SD 4.96 years; n=327, 68.1% male). Study 2 included 565 adults (mean age 25, SD 5.55 years; n=245, 43% male). Exploratory factor analysis revealed a 3-factor model of withdrawal symptoms: (1) motivational and cognitive symptoms, (2) affective symptoms, and (3) physical symptoms, explaining 54% of the variance. Confirmatory factor analysis confirmed adequate model fit (*χ*^2^_227_=887.8; *P*<.001; comparative fit index=0.91; Tucker‐Lewis index=0.90; root-mean-square error of approximation=0.072). The GWSQ demonstrated high internal consistency, with Cronbach α ranging from 0.89 (motivational and cognitive symptoms) to 0.90 (affective symptoms and physical symptoms). Correlations with related constructs (Internet Gaming Disorder Scale-Short Form [IGDS9-SF], Patient Health Questionnaire-9 items, and Generalized Anxiety Disorder-7 items) confirmed convergent validity with moderate associations (eg, IGDS9-SF: *r*=0.48, 95% CI 0.32-0.61) and discriminant validity, and normative data (sten scores) were established for the general population.

**Conclusions:**

The GWSQ is the first validated multidimensional tool specifically designed to assess withdrawal symptoms in GD, representing a conceptual and methodological innovation. It addresses critical gaps in GD diagnosis and research by capturing a broader spectrum of symptoms beyond affective distress. The questionnaire’s tripartite structure provides a framework for advancing theoretical models and informing etiological studies of GD. The GWSQ offers a robust measure for clinical research and enables differentiated assessment of symptom clusters. In real-world contexts, it can serve as a reliable patient-reported outcome tool in forthcoming clinical trials of GD interventions, enabling precise monitoring of treatment effects**.** Nevertheless, given the nonclinical sample, further cultural validation and studies involving clinical populations are required.

## Introduction

### Background

Playing video games is an increasingly popular form of leisure activity with more than 3.3 billion gamers worldwide [1]. The popularity of games is growing with technological developments and easier access to immersive virtual worlds [2]. Although for most people gaming is a nonproblematic recreational activity, for a minority of users it has become poorly controlled and has negative psychosocial consequences [3-5]. According to different sources, disordered gaming impacts 3%-17% of the population, depending on the geographic region [6-9].

In 2013, for the first time, internet gaming disorder (IGD) was recognized as a potential new disorder and included as a “condition for further study” in the *DSM-5* (*Diagnostic and Statistical Manual of Mental Disorders, Fifth Edition*) [10]. The positioning of IGD in Section III of the *DSM-5* was intended to encourage researchers to conduct further studies to determine whether the disorder should be included in the next generation of the *Diagnostic and Statistical Manual of Mental Disorders*. The criteria used to diagnose IGD include preoccupation, withdrawal symptoms, tolerance, loss of control, giving up other activities, continuation of gaming despite negative consequences, deception, using games to escape, and losing relationships/career/education because of gaming. More recently, in 2019, gaming disorder (GD) was recognized as a mental disorder and listed as a “disorder due to addictive behaviors” in the *International Classification of Diseases, Eleventh Edition* (*ICD-11*) [11]. Notably, the World Health Organization has taken a more conservative approach and proposed GD criteria characterized by 3 obligatory features (loss of control, increasing priority given to games, and continuation of gaming despite negative consequences) associated with clinically significant impairment. Despite the scientific debate on diagnostic criteria for gaming-related disorders and intensive research in the field, the most recent version of the *DSM-5 (DSM-5 TR [Diagnostic and Statistical Manual of Mental Disorders, Fifth Edition, Text Revision]*) does not include an updated definition of IGD [12].

The presence of the withdrawal symptoms criterion is the main difference between the diagnosis of GD proposed in *DSM-5* and *ICD-11* [13]. Withdrawal symptoms, according to the definition proposed by the American Psychological Association (APA) in the *DSM-5*, refer to symptoms such as anxiety, irritability, and sadness that occur when gaming is not possible [10]. This implies that withdrawal is not necessarily physiological in nature. Since the definition extends only to the emotions a gamer may experience, this criterion is one of the most criticized aspects of the entire GD diagnosis [13-16]. It has been argued that withdrawal in the context of GD lacks a neurobiological basis, as there is no psychoactive substance interaction as in substance addictions [16]. Furthermore, it was also debated whether the withdrawal criterion is capable of distinguishing between passionate engagement in gaming and pathological gaming [14,17,18]. Nevertheless, recent studies have shown that withdrawal plays a central role in GD symptomatology and is a valid criterion for distinguishing players with GD from healthy gamers [19-23]. These studies are in line with the traditional division of addiction symptoms into core and peripheral ones, where withdrawal is classified as a core symptom that distinguishes addicted gamers from highly engaged gamers [24,25].

### Current Perspectives on Withdrawal Symptoms in GD

Since withdrawal plays a crucial role in GD symptomatology, it requires an in-depth understanding, including precise characterization and methods for assessment. Based on the APA’s 2013 definition, which describes withdrawal symptoms as sadness, anxiety, and irritability, this characterization appears insufficiently specific and lacks comprehensive detail. First, the withdrawal criterion should incorporate a defined time frame that specifies when symptoms are expected to emerge. Petry et al [15] emphasize that withdrawal symptoms should be carefully differentiated from emotional reactions that result from an external interruption or prevention of gaming. Genuine withdrawal manifests itself as distress lasting several hours to days following a cessation of gaming, distinguishing it from the immediate emotional responses triggered by external factors, such as a person forcibly stopping the game [14,15,26]. To distinguish negative emotions related to withdrawal from those caused by other factors, the assessment of withdrawal symptoms should be specified in such a way that symptoms disappear once the gamer plays again [14].

Continuing further, withdrawal may be more complex and multidimensional than initially described. Starzec et al [27], in their scoping review on the conceptual definition of withdrawal, which analyzed 3701 publications from 2018 to 2024, confirmed the presence not only of the affective component, but also of the cognitive, behavioral, physiological, and neurological dimensions. Based on three sources: (1) the specific definition provided by the authors, (2) the symptoms identified in the measurement tools through withdrawal check items, and (3) descriptions of participants’ experiences of withdrawal, the review authors significantly expanded the list of abstinence symptoms in GD. In the affective component, they list symptoms such as anxiety, feeling depressed, sadness, feelings of stress, fear, restlessness, anger, impatience, nervousness, feelings of unhappiness, emptiness, irritability, frustration, and boredom. The physiological component includes symptoms such as craving, decreased hunger, decreased eating, stomach problems, sweating, increased sleeping, headaches, and muscle pain. Furthermore, the cognitive component of withdrawal symptoms was extended to include difficulty concentrating, preoccupation, and intense thoughts of gaming. Behavioral symptoms (eg, inability to do other things and inability to relax) were also identified, as well as neurological symptoms (eg, functional connectivity was observed between the striatum and thalamus) [27]. These findings highlight the multifaceted nature of withdrawal in GD, underscoring the need for comprehensive assessment methods that capture its full complexity.

Furthermore, the methods of measuring withdrawal have also been examined in detail. A scoping review of the role of withdrawal in contemporary GD research provided further insight into how these symptoms are operationalized [27]. One of the most commonly used methods for assessing withdrawal involves self-report instruments, but other approaches, such as clinical interviews and physiological and neurophysiological measures, have also been applied [27,28]. While self-report instruments are widely used for assessing withdrawal symptoms, they have several limitations. First of all, in GD research, withdrawal is examined as a single questionnaire item across the IGD screening tools. This is the case with the Internet Gaming Disorder Scale-Short Form (IGDS9-SF), where withdrawal symptoms are assessed based on the question: “Do you feel more irritability, anxiety or even sadness when you try to either reduce or stop your gaming activity?” [29]. A similar approach to the withdrawal assessment was used in the Internet Addiction Test [30], Compulsive Internet Use Scale [31], 10-Item Internet Gaming Disorder Test [32], Chen Internet Addiction Scale-Gaming Version [33], Internet Gaming Disorder Criteria Checklist [15], and Screening Test for Problematic Gaming [34]. Additionally, several withdrawal screening tools have been developed based on withdrawal symptoms in substance dependence; however, these symptoms may differ from those found in behavioral addictions. Examples of this include the Internet Gaming Withdrawal Scale [35], which is a modified version of the Penn Alcohol Craving Scale [36]. Likewise, the Abstinence Symptoms Checklist, adapted to the gaming context by Giordano et al [37], was originally designed for cocaine withdrawal symptoms. To the best of our knowledge, to date, there is no withdrawal assessment tool that takes into account the specificity of GD and is directly designed for behavioral addictions, which may differ from substance use disorders. Moreover, there is no standardized tool that captures the multidimensionality of withdrawal, not only from an affective dimension, but also from a cognitive, behavioral, and physiological perspective.

The development of a more refined and comprehensive withdrawal questionnaire is essential for advancing both research and clinical practice. Current research highlights that withdrawal symptoms, including cravings and mood disturbances, are not only key indicators of GD but also predictors of more severe cases [38]. A specialized questionnaire would be crucial in accurately differentiating between normal and pathological gaming behavior, making it vital for early detection and monitoring the progression of the disorder. Moreover, such a tool would be instrumental in capturing the multidimensionality of withdrawal, considering its affective, cognitive, behavioral, and physiological dimensions, which are often overlooked in existing assessments. This would allow for a more nuanced understanding of withdrawal, helping clinicians develop tailored treatment and prevention strategies. Furthermore, research supports the claim that GD is a major risk factor for the development of other mental diseases, such as depression and anxiety disorders [19,39,40]. This indicates that a comprehensive evaluation of withdrawal symptoms (eg, mood swings, sleep disturbances, and concentration difficulties) could help detect emerging mood disorders at an early stage. Identifying these symptoms early would allow for the implementation of preventive interventions, potentially averting the escalation of both gaming-related issues and cooccurring mental health problems.

### Overview of the Work

To achieve the objectives of this research, we implemented a structured, multistage approach. The development process began with a reanalysis of data from a scoping literature review, aimed at identifying measurement methods and withdrawal symptoms associated with GD. These findings guided the construction of the Gaming Withdrawal Symptoms Questionnaire (GWSQ). Next, during the validation process, we conducted 2 empirical studies. Study 1 involved an exploratory factor analysis (EFA) to identify the underlying structure of the questionnaire, along with an assessment of internal consistency. Study 2 used a confirmatory factor analysis (CFA) to validate this structure, alongside evaluations of convergent and divergent validity to ensure the robustness of the GWSQ as a psychometric tool. Finally, the normalization phase focused on establishing interpretation guidelines, including the evaluation of sten standards, to facilitate the practical application of the GWSQ in research and clinical settings.

### Aim of the Study

In order to advance research and further the scientific understanding of gaming-related disorders, we aim to develop the first comprehensive withdrawal inventory (covering all symptoms identified in the literature) to evaluate the core criteria for GD [27]. This is especially important due to the absence of precise diagnostic criteria for withdrawal and limited comparability between the IGD and the GD criteria. This is a crucial step in GD research in order to provide empirical data related to the diagnostic properties and efficacy of withdrawal criteria. Consequently, it will influence not only GD research but also clinical practice.

## Methods

### Development of GWSQ

**Figure 1:**
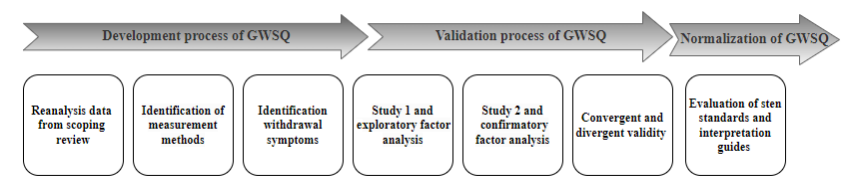
Methodological framework for the Gaming Withdrawal Symptoms Questionnaire (GWSQ): development, validation, and normalization. This figure summarizes the 2-study design used for the conceptualization and exploratory factor analysis (study 1; n=480) and the psychometric validation via confirmatory factor analysis (study 2; n=565) of GWSQ. The studies were conducted among a predominantly Polish-speaking sample of gamers.

The development and validation of the GWSQ, which involved 2 cross-sectional phases (Study 1: EFA, Study 2: CFA), followed recommendations for transparent and complete reporting of instrument development studies (Figure 1). Specifically, the research adhered to established psychometric standards, including the principles outlined in the COSMIN (Consensus-Based Standards for the Selection of Health Measurement Instruments) guidelines [41] and the STROBE (Strengthening the Reporting of Observational Studies in Epidemiology) statement [42] for cross-sectional studies. The completed checklists for these reporting guidelines have been provided in Multimedia Appendix 1.

The GWSQ was developed and validated in the English language. The primary phase of instrument creation focused on establishing content validity and generating a comprehensive item pool.

1. Content domain definition: the initial item pool was generated through a scoping literature review aimed at systematically identifying all available empirical measures and conceptual definitions of gaming withdrawal symptoms. This systematic review established the content domain of the GWSQ, ensuring it was comprehensive and grounded in existing research and clinical practice. A detailed description of the review methodology, selection process, and results regarding the operational and conceptual definition of gaming withdrawal symptoms is presented in a previous publication by the authors [27].
2. Initial item pool: the synthesis of the scoping review findings resulted in a pool of 47 distinct withdrawal symptoms. These symptoms were formulated into preliminary questionnaire items, with each item using a 5-point Likert scale (1=strongly disagree to 5=strongly agree). The initial pool of items was designed to reflect 4 preliminary theoretical dimensions: affective, cognitive, behavioral, and physical symptoms.

Table 1 presents the specific symptoms identified and used for item generation.

**Table 1:** Identified gaming withdrawal symptoms based on a scoping review of existing measurement tools used to assess withdrawal in the context of gaming disorder. The table groups symptoms into preliminary domains (affective, cognitive, behavioral, and physical). “n” indicates the number of tools in the scoping review [27] that contained the specific symptom. This step informed the item pool generation for the Gaming Withdrawal Symptoms Questionnaire development.

Symptom			Value, n
**Affective symptoms**			
	Irritability	13	
	Anxiety	11	
	Sadness	6	
	Stress	2	
	Fear	1	
	Feeling bad	1	
	Restlessness	8	
	Feeling moody	2	
	Anger	3	
	Feeling depressed	8	
	Impatience	2	
	Feeling upset	1	
	Feeling uneasy	1	
	Frustration	3	
	Nervousness	1	
	Feeling lethargic	1	
	Dysphoria	2	
	Unsatisfaction	1	
	Boredom	2	
	Distress	1	
	Emptiness	1	
**Cognitive symptoms**			
	Thoughts about gaming	2	
	Difficulty concentrating	1	
	Preoccupation with gaming	2	
**Behavioral symptoms**			
	Inability to experience pleasure	2	
	Lack of pleasure	1	
	Disinclination for activity	2	
	Inability to do other things	1	
	Inability to identify activities to do	1	
	Attempts to find games to play	1	
	Lack of motivation	1	
	Inability to relax	1	
**Physical symptoms**			
	Urge	2	
	Craving	2	
	Decreased hunger	1	
	Decreased eating	1	
	Increased eating	1	
	Headache	1	
	Stomach problems	1	
	Sweating	1	
	Insomnia	1	
	Increased sleeping	1	
	Increased dreaming	1	
	Muscle pain	1	
	Chills, tremors/shakes, and twitching	1	
	Backaches or other physical discomfort	1	
	Fatigue	1	

Given that the 47 items were systematically derived and operationalized directly from the empirical and clinical literature via the published scoping review, the authors used large-scale factor analysis for subsequent item refinement. The EFA performed in Study 1 thus served as the initial robust empirical method for item purification and scale construction. This statistical approach was used to reduce the initial pool of 47 items, verify the underlying latent factor structure, and ensure the construct validity and internal consistency of the emerging GWSQ subscales. The subsequent CFA in Study 2 provided final validation of the structure.

### Study 1: Exploratory Validation

#### Participants and Procedure

Participants were recruited through advertisements on gaming-related social media groups using convenience and snowball-sampling methodologies. The posts included information about the study, that is, an invitation to participate, a description of the topic of the study, an estimated time to complete the survey, an assurance of anonymity, an opportunity to withdraw at any time, assurance of confidentiality, and an explanation of the purpose of data use. Data collection was conducted through an online survey hosted on Qualtrics [43], which was performed between January and February 2024. Participation was entirely voluntary, and no financial compensation was offered to participants.

Before starting the questionnaire, participants provided their informed consent to continue and take part in the study. The eligibility criteria required participants to be 18 years of age or older and actively involved in video gaming. Participants became eligible to take part in the study upon responding to the following screening question: “Have you played any video games in the past 12 months (yes/no)?” This criterion was chosen in accordance with previous studies [13,44,45] that demonstrated its effectiveness in identifying individuals with gaming experience. Only participants who provided a confirming answer were allowed to participate in the study. A total of 21 participants were excluded based on this inclusion criterion. To qualify for analysis, participants had to pass at least 1 attention check. The attention check questions were designed to ensure that participants were reading the questions carefully and engaging with the task. Specifically, participants were asked to select a predetermined answer. Finally, the results of 101 participants were excluded on the basis of a negative answer to this question. A total of 602 people took part in the study. Due to the unsuccessful screening question (n=21) and the failure of the attention check (n*=*101), 480 results were included in the analysis. The minimum required sample size for the EFA was determined based on the established psychometric guideline of a minimum 10:1 participant-to-item ratio (47 items), necessitating a sample of 470 participants [46,47].

#### Measures

##### Sociodemographic Information

Sociodemographic data included participants’ gender, age, and nationality. If participants entered their age as <18 years, they were informed that they could not participate further in the study. Participants aged <18 years were excluded from the study due to potential concerns about the influence of external factors, such as parental restrictions on gaming behavior.

##### GWSQ Tool

The GWSQ is an assessment tool designed to measure the intensity of withdrawal symptoms. The GWSQ items were developed in the English language to ensure the scale’s content was universal and applicable to international studies. The GWSQ includes 47 items reflecting all symptoms identified during the systematic review process. The items are organized conceptually into 4 components: affective (eg, sadness and irritability), behavioral (eg, attempts to find games to play and inability to relax), cognitive (eg, concentration problems and thoughts about gaming), and physiological (eg, headaches and sweating). Respondents were instructed to indicate the intensity of those feelings or states they experienced in situations when they desired to play games but were unable to do so. All items are rated on a 5-point Likert scale: 1 (“not at all”), 2 (“slightly intense”), 3 (“moderately intense”), 4 (“intense”), and 5 (“extremely intense”). The psychometric properties of the final scale, including its internal consistency reliability (Cronbach α), are detailed in the Results section.

##### Gaming Involvement Scale

The Gaming Involvement Scale (GIS) is a self-report tool to measure involvement in video game-related activities across six different indications: (1) playing video games, (2) watching game-related video, (3) thinking about video games, (4) reading game-related content, (5) talking about video games, and (6) considering game-related purchases. The English version of the scale was administered to all participants. Participants were asked to estimate the amount of time (in minutes) they dedicated to these activities on an average workday and an average weekend day. The scale was developed in previous studies by the team [48]. The internal consistency was good (Cronbach α=0.80).

##### IGDS9-SF Measurement

The IGDS9-SF [29] is a psychometric measurement adapted from the 9 IGD criteria according to the *DSM-5* classification [10]. The English version of the tool was used. The tool consists of 9 items that are rated on a 5-point Likert scale: 1 (“never”), 2 (“rarely”), 3 (“sometimes”), 4 (“often”), and 5 (“very often”). Total scores can range from 9 to 45, with higher scores indicative of higher levels of disordered gaming. An example of an item is: “Do you feel more irritability, anxiety or even sadness when you try to either reduce or stop your gaming activity?” The internal consistency was good (Cronbach α=0.84).

#### Statistical Approach

All statistical analyses were performed using SPSS Statistics software (version 29; IBM Corp). Prior to data analysis, the completeness of the GWSQ items was assessed in Study 1. No missing data were observed, which precluded the need for applying the missing completely at random test or handling missing-data techniques such as multiple imputation. Then, the statistical analysis of the collected data included descriptive statistics of the main sample’s characteristics. Subsequently, to examine the underlying structure of the GWSQ, we used EFA. Given that the data were not approximately normally distributed, we used the principal-axis method as the extraction method. To determine the number of factors to fit the data, we used scree-plot inspection combined with the Kaiser rule. Due to the high likelihood of between-item correlations, oblimin rotation was used for extraction. Items with a loading of 0.4 or higher were included in the factor, and if items were loading on 2 factors, they were included in the factor with the highest loading.

### Study 2: Confirmatory Validation

#### Participants and Procedure

The inclusion criteria for participation in Study 2 were the same as in the previous one (being 18 years of age or older and playing video games). If the respondent did not meet the age and gaming criteria, they were not allowed to participate in the study. The instructions indicated that the participation was anonymous, voluntary, and that the data were collected for research purposes only. Respondents to Study 2 were sought via gaming-related social media (Facebook, Reddit, Discord, and Steam Community), and all participants had not participated in Study 1. Data collection was performed using an online survey hosted on the Qualtrics platform [43] and took place between November and December 2024. The survey included sociodemographic questions and a set of questionnaires. In order to control the quality of the data, 2 attention check questions were used in the study. From the initial sample of 590, we excluded participants who did not play games (n=4) or failed the double attention check (n=21). Subsequently, a total of 565 participants were included in the final analysis.

#### Measures

##### Sociodemographic Information

The study collected demographic data, which included gender, age, and nationality of participants.

##### GWSQ Tool

The GWSQ consists of 23 items. This version is based on the results of an EFA conducted in Study 1. The English version of the scale was used. Responses were given on a 5-point Likert scale, ranging from 1 (“not at all”) to 5 (“extremely intense”). Examples of items include: “Anxiety,” “Irritability,” and “Thoughts about gaming.”

##### IGDS9-SF Tool

The IGDS9-SF [49] is a short, standardized psychometric tool for the assessment of IGD. The scale contains 9 criteria that form the basis for a possible diagnosis according to APA’s guidelines [10]. The participants answered 9 questions about their gaming behavior and experiences over the past 12 months. Cronbach α coefficient for the questionnaire was 0.74.

##### Patient Health Questionnaire-9

The Patient Health Questionnaire-9 items (PHQ-9) [50] is a useful tool for screening for depression. It was used to measure the level of depressive symptoms of study participants. This scale is based on the criteria for the disorder, as contained in the *Diagnostic and Statistical Manual of Mental Disorders, Fourth Edition* [51]. The answers of the questionnaire are rated on a 4-point Likert scale, ranging from 0 (“not at all”) to 3 (“nearly every day”). An example of an item is: “Little interest or pleasure in doing things.” In this study, the internal consistency was high (Cronbach α coefficient for the questionnaire was 0.87).

##### Generalized Anxiety Disorder-7 items

The Generalized Anxiety Disorder-7 items (GAD*-*7) [52] is a brief self-report to identify the presence of anxiety symptoms in participants. All participants completed the English version of the scale. The scale uses a 4-point Likert scale, ranging from 0 (“not at all”) to 3 (“nearly every day”). An example of an item is: “Feeling nervous, anxious, or on edge.” The internal consistency of the questionnaire in this study was high (Cronbach α=0.89).

##### GIS Questionnaire

The GIS [48] is a questionnaire designed to assess the extent of involvement in gaming activities. Participants received the English version of the scale. The participant was asked to answer a question regarding how they spent their time on particular video game–playing activities. The respondent was required to determine the number of minutes spent on 6 activities during both working and weekend days. These activities included playing video games, contemplating playing video games, reading or watching tutorials, reviews, theory, or additional lore related to video games, watching video game streams and gameplay videos (including e-sports games), talking or writing about video games, and purchasing additional gaming devices or gadgets. In this study, the scale demonstrated acceptable internal consistency (Cronbach α=0.79).

##### Gaming Motivation Inventory

The Gaming Motivation Inventory (GMI) [53] was included in the study to control the level of motivation to play video games among participants. The English version of the questionnaire was given to all participants. The selected subscales were “Recreation” and “Introjected regulation.” The “introjected regulation” subscale includes items such as “because I must play to feel good about myself” and “because otherwise I would feel bad about myself,” reflecting extrinsic motivation driven by internal pressures or guilt. This subscale was hypothesized to have strong convergence with withdrawal symptoms, as both are indicative of problematic gaming behavior. On the other hand, the “Recreation” subscale, which includes items such as “because it is fun” and “to relax,” represents intrinsic motivation tied to the enjoyment and leisure aspects of gaming. This subscale was expected to show weaker or no correlation with withdrawal symptoms, serving as a measure of divergent validity. A total of 6 GMI items were used in the survey. The questionnaire used a 7-point Likert scale ranging from 1 (“It does not correspond at all”) to 7 (“It corresponds exactly”). An example of an item from the “Introjected regulation” subscale is “because I must play to feel good about myself.” The respondent was given the opportunity to select one of the options from 1 “It does not correspond at all” to 7 “It corresponds exactly.” The internal consistency of the scale was good (Cronbach α=0.81).

##### Satisfaction With Life Scale

The Satisfaction With Life Scale (SWLS) [54] focuses on happiness in hedonistic terms (occurrence of positive emotions, nonoccurrence of negative emotions, and satisfaction with the situation). Participants received the English version of the scale. The scale contains 5 items. The 7-point Likert scale ranges from 1 (“strongly disagree”) to 7 (“strongly agree”). An example of an item is “In most ways my life is close to my ideal.” Once all scores have been aggregated, the resulting total score provides an indication of overall satisfaction with life. The score ranges from 5 to 35 points. In this study, the internal consistency was high (Cronbach α coefficient for the questionnaire was 0.87).

##### Psychological Well-Being Scale

The Psychological Well-Being Scale [55] measures eudaimonic happiness, focusing on self-actualization, self-acceptance, personal development, and mastery over the environment. All participants responded to the English version of the scale. The scale consists of 18 items. A 7-point Likert scale was used in the questionnaire, ranging from 1 (“strongly disagree”) to 7 (“strongly agree”). An example of an item is “I like most parts of my personality.” In this study, the internal consistency was high (Cronbach α coefficient for the questionnaire was 0.85).

#### Statistical Approach

Similar to Study 1, an assessment of data completeness for all GWSQ items in Study 2 revealed no missing data. Consequently, neither the Little missing completely at random test nor any missing-data handling techniques were required for subsequent analyses. Statistical analyses were performed using SPSS Statistics software (version 29) and the *lavaan* package in R software (version R-4.4.2; R Foundation for Statistical Computing). First, descriptive statistics of the main sample’s characteristics were examined using SPSS. Subsequently, CFA was conducted using the *lavaan* package in R software. Maximum likelihood estimation was used. The hypothesized model was a 3-factor model as described above. The model was assessed using the chi-square with respective *P* values (*P*>.01), root-mean-square error of approximation (<0.06), comparative fit index (>0.95), Tucker‐Lewis index (>0.95), and standardized root-mean-square residual (<0.08) [56].

### Ethical Considerations

This study was conducted in accordance with the principles of the Declaration of Helsinki. The approval was granted by the Research Ethics Committee at the Institute of Applied Psychology of the Jagiellonian University (opinion number 102/2021). The approval date was July 19, 2021. Informed consent was obtained from all individual participants included in the study. Privacy and confidentiality were rigorously protected, as the study was anonymous. Participation was voluntary, and participants received no financial compensation. No images or supplementary materials included in this manuscript allow for the identification of individual participants.

## Results

### Study 1: Exploratory Validation

#### Descriptive Statistics

Descriptive statistics for the sample demographics, gaming engagement, and severity of GD symptoms are presented in Table 2. The total sample for Study 1 consisted of 480 participants who were predominantly male (n=327, 68.1%) with a mean age of 23 (SD 4.96) years. The sample was predominantly Polish (n=473, 98%), which necessitates caution regarding generalizability.

**Table 2:** Demographic and gaming-related information of the sample (Study 1).

Characteristic		Value (n=480)
**Gender, n (%)**		
	Male	327 (68.1)
	Female	138 (28.7)
	Nonbinary	12 (2.5)
	Refused to state	3 (0.6)
Age (years), mean (SD; range)		23 (4.96; 18-53)
**Nationality, n (%)**		
	Poland	473 (98)
	United Kingdom	4 (0.8)
	Germany	1 (0.2)
	Australia	1 (0.2)
	Poland-Germany	1 (0.2)
**Gaming engagement (hours), mean (SD)**		
	Playing video games	13.6 (16.88)
	Watching video game streams and gameplay videos	8.27 (9.17)
	Reading or watching game-related content	7.11 (11.63)
	Talking or writing about video games	7.15 (10.13)
	Considering buying additional content or collectibles related to games	2.65 (6.55)
**Severity of GD symptoms, mean (SD)**		
	IGDS9-SF^a^	17.27 (6.02)

^a^IGDS9-SF: Internet Gaming Disorder Scale-Short Form.

Analysis of gaming engagement showed that participants spent an average of 23 (SD 16.39) hours per week actively playing video games. Beyond primary gameplay, thinking about video games was the most time-consuming secondary activity, while the least amount of time was dedicated to considering the purchase of additional content.

The severity of GD symptoms, as measured by the IGDS9-SF, demonstrated a mean score of 17.27 (SD 6.02). The median score of 16 (IQR 13-20) and a mode of 11 suggest a positive skew in the distribution of IGD symptoms within this cohort.

#### EFA Method

An initial analysis was performed to verify the justification for subjecting the collected material to factor analysis. Kaiser-Meyer-Olkin measure of sampling adequacy was 0.94. The Bartlett sphericity test result was as follows: *χ*^2^_1128_=12468.1; *P*<.001, which indicates the validity of performing a factor analysis due to the heterogeneous structure of the questions.

Following initial exploration, the principal-axis method was conducted on the full item set. Based on the Kaiser criterion (*l*>1), 9 factors were initially extracted, explaining 62% of the common variance. Items with factor loadings below 0.40 were systematically excluded from the analyses. A total of 24 items did not meet the factor loading threshold (≥.40) and were removed from the scale. After the removal of these items, 23 items remained for further analysis. Consequently, the initial 9-factor solution was reduced to a 3-factor model.

To confirm the dimensionality of the retained items, the EFA was rerun using the principal-axis method with an oblimin rotation (due to expected correlations between factors). The final 3-factor solution was extracted, which explained 54% of the common variance. The complete factor loading matrix for this structure is shown in Table 3.

**Table 3:** Factor structure of the Gaming Withdrawal Symptoms Questionnaire derived from exploratory factor analysis (EFA; study 1). The EFA used principal-axis method with oblimin rotation and Kaiser normalization on a sample of 480 gamers (n=473, 98% Polish-speaking participants). Data were collected online between January and February 2024.

	Factor 1^a^	Factor 2^b^	Factor 3^c^
Inability to do other things	0.84^d^	–0.01	0.03
Inability to identify activities to do	0.84^d^	–0.04	–0.07
Disinclination for activity	0.78^d^	–0.05	0.05
Inability to relax	0.77^d^	0.07	–0.06
Difficulty concentrating	0.69^d^	0.09	0.07
Lack of motivation	0.68^d^	0.09	0.18
Preoccupation with gaming	0.58^d^	0.14	0.09
Inability to experience pleasure	0.54^d^	0.05	0.26
Frustration	0.03	0.84^d^	–0.08
Anger	–0.10	0.82^d^	0.13
Irritability	–0.08	0.76^d^	0.05
Impatience	0.10	0.69^d^	–0.15
Feeling upset	0.16	0.69^d^	–0.09
Nervousness	0.05	0.66^d^	0.27
Feeling bad	0.34	0.46^d^	0.00
Sweating	–0.17	0.14	0.79^d^
Muscle pain	0.09	–0.12	0.70^d^
Chills, tremors/shaking, and twitching	–0.05	0.09	0.69^d^
Stomach problems	0.04	–0.05	0.69^d^
Headaches	0.07	–0.04	0.68^d^
Backaches or other physical discomfort	0.06	0.02	0.65^d^
Decreased eating	0.20	–0.04	0.46^d^
Increased dreaming	0.20	–0.03	0.45^d^

^a^Factor 1: motivational and cognitive withdrawal symptoms.

^b^Factor 2: affective withdrawal symptoms.

^c^Factor 3: physical withdrawal symptoms.

^d^Values indicate the highest factor loading for each item (primary factor loading).

Consistent with the use of the oblimin oblique rotation, the 3 extracted factors were correlated. The interfactor correlations ranged from 0.19 to 0.49. Specifically, the correlation between the motivational and cognitive factor (F1) and the affective factor (F2) was ϕ=0.39; between F1 and the physical factor (F3) was ϕ=0.49; and between F2 and F3 was ϕ=0.19. These interfactor correlations support the existence of a higher-order latent construct (gaming withdrawal symptoms) while confirming that the 3 dimensions are distinct, as no correlation exceeded the threshold of ϕ=0.85, which typically suggests redundant factors.

In connection with the analysis of items included in individual subscales, the following names were chosen for the extracted components:

- Factor 1 (Motivational and Cognitive Withdrawal Symptoms): the subscale includes the following 8 items connected with an individual’s involvement in various activities: “Inability to do other things,” “Inability to identify activities to do,” “Disinclination for activities,” “Inability to relax,” “Lack of motivation,” “Difficulty concentrating,” “Preoccupation with gaming,” and “Inability to experience pleasure.” The factor loading values for the given items range from 0.54 to 0.84. The subscale demonstrated high internal consistency with Cronbach α=0.90.
- Factor 2 (Affective Withdrawal Symptoms): the factor contains the following 7 items related to heightened emotional states: “Frustration,” “Anger,” “Irritability,” “Nervousness,” “Impatience,” “Feeling upset,” and “Feeling bad.” The factor loading values for the given items range from 0.46 to 0.84. The internal consistency of the subscale was high (Cronbach α=0.86).
- Factor 3 (Physical Withdrawal Symptoms): the subscale includes the following 8 items related to experienced physiological symptoms: “Sweating,” “Chills, tremors/shaking, and twitching,” “Headaches,” “Muscle pain,” “Stomach problems,” “Backaches or other physical discomfort,” “Decreased eating,” “Increased dreaming.” The factor loading values for the given items range from 0.45 to 0.79. Factor 3 demonstrated good reliability, assessed using Cronbach α (Cronbach α=0.80).

The initial EFA confirmed the validity of subjecting the collected data to a factorial structure examination. Although an initial 9-factor solution was extracted following the Kaiser criterion, a refinement process led to the identification of a 3-factor model that explained 54% of the total variance. This model includes Motivational and Cognitive Withdrawal Symptoms, Affective Withdrawal Symptoms, and Physical Withdrawal Symptoms, all demonstrating good internal consistency (Cronbach α ranging from 0.80 to 0.90). The structure highlights the complexity of gaming withdrawal, extending beyond emotional distress to include cognitive difficulties and somatic complaints. To further validate the identified 3-factor model, a CFA was conducted in the next stage of the study. Additionally, convergent and divergent validity and the evaluation of scale standards were performed. To perform such analyses, Study 2 applied additional measurement tools: the PHQ-9 [50], the GAD-7 [52], the SWLS [54], the Psychological Well-Being Scale [55], and the GMI [53].

### Study 2: Confirmatory Validation

#### Descriptive Statistics

Descriptive statistics characterizing the sample’s demographics and gaming engagement patterns for the total sample (N=565) are presented in Table 4. The sample exhibited a concentration of Polish participants, accounting for the vast majority of respondents. Other nationalities were present in very low numbers. Regarding gender distribution, the proportion of female participants was slightly higher than that of males. A small number of individuals identified as nonbinary or declined to specify their gender. Participant ages ranged from 18 to 45 years.

**Table 4:** Demographic and gaming-related information of the sample (study 2).

Demographic			Value (n=565)
**Gender, n (%)**			
	Male	245 (43)	
	Female	294 (52)	
	Nonbinary	13 (2)	
	Refused to state	10 (1.77)	
Age (years), mean (SD; range)			25 (5.55; 18-45)
**Nationality, n (%)**			
	Poland	545 (96.4)	
	United Kingdom	4 (0.7)	
	Nederland	4 (0.7)	
	United States	2 (0.35)	
	Canada	1 (0.17)	
	China	1 (0.17)	
	France	1 (0.17)	
	India	1 (0.17)	
	Italy	1 (0.17)	
	Malaysia	1 (0.17)	
	Scotland	1 (0.17)	
	Singapore	1 (0.17)	
	Thailand	1 (0.17)	
	Vietnam	1 (0.17)	
**Gaming engagement (hours), mean (SD)**			
	Playing video games	9.25 (7.5)	
	Watching video game streams and gameplay videos	3.65 (6.5)	
	Reading or watching game-related content	1.86 (3.08)	
	Talking or writing about video games	1.73 (3.83)	
	Considering buying additional content and collectibles related to games	0.43 (1.3)	
**Severity of GD symptoms, mean (SD)**			
	IGDS9-SF^a^	18.64 (5.79)	

^a^IGDS9-SF stands for the Internet Gaming Disorder Scale-Short Form.

Analysis of self-reported weekly gaming-related behaviors based on the GIS indicated that participants spent the most time actively playing video games. The next highest time commitment was devoted to watching video game streams and gameplay videos. Other related activities, such as reading game-related content or talking about video games, constituted significantly less weekly time commitment. The least amount of time was spent on considering the purchase of additional game content.

Descriptive statistics were also computed for the IGDS9-SF to characterize self-reported symptom severity.

#### CFA Results

**Figure 2:**
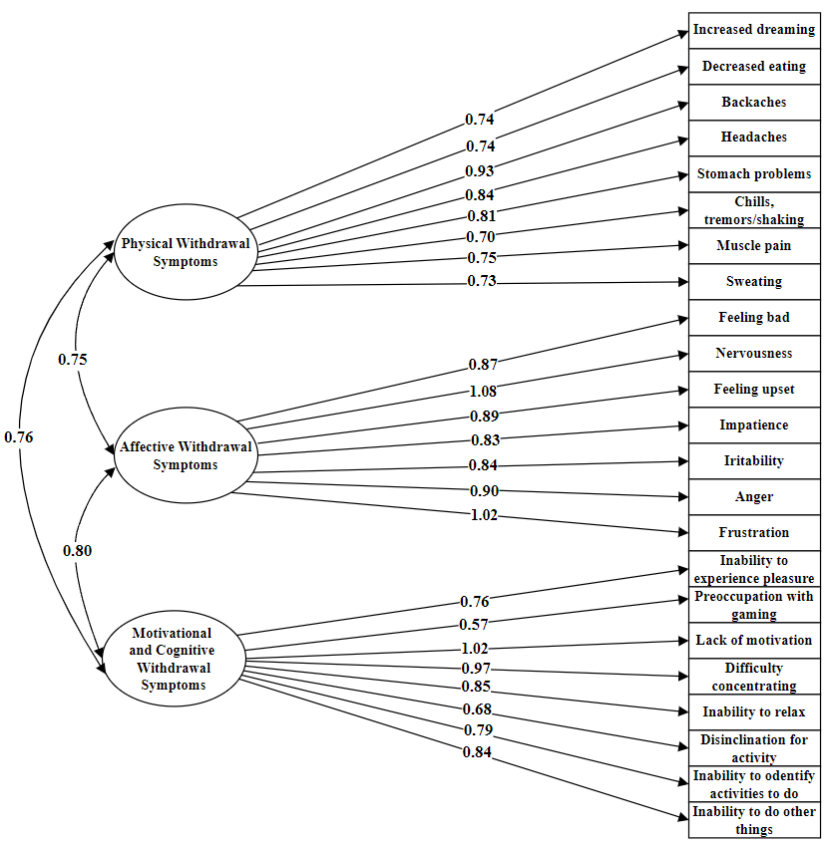
Three-factor model of the Gaming Withdrawal Symptoms Questionnaire (GWSQ). The figure illustrates the final confirmatory factor analysis structure (motivational and cognitive, affective, and physical) of GWSQ. The model was tested on the study 2 sample (n=565 predominantly Polish-speaking participants). This figure displays the 3-factor structure of GWSQ, including standardized factor loadings and interfactor correlations.

The hypothesized model demonstrated an acceptable, though not perfect, fit to the data. CFA conducted on an independent sample of 565 gamers (n=545, 96.4% Polish-speaking participants) showed the following fit indices: *χ*^2^_227_=887.8; *P*<.001; comparative fit index=0.91; Tucker-Lewis index=0.90; root-mean-square error of approximation=0.072 (90% CI 0.067-0.077); standardized root-mean-square residual=0.049. The model was estimated using maximum likelihood estimation. All factor loadings were statistically significant (*P*<.001). The standardized factor loadings are presented in Figure 2.

#### Convergent and Divergent Validity

To assess the convergent and discriminant validity of the GWSQ, Pearson correlations were conducted with selected measures assessing related and distinct constructs. The results are presented in Tables 5 and 6.

**Table 5:** Correlations between the Gaming Withdrawal Symptoms Questionnaire (GWSQ) and measures of convergent validity. Three decimal places were reported for enhanced precision, particularly to better differentiate between closely related values.

Variable	GWSQ, *r* (95% CI)
IGDS9-SF^a^	0.476^b^ (0.321-0.606)
PHQ-9^c^	0.479^b^ (0.321-0.612)
GAD-7^d^	0.415^b^ (0.246-0.560)
GMI^e^ (introjected regulation)	0.324^b^ (0.137-0.488)

^a^IGDS9-SF: Internet Gaming Disorder Scale-Short Form.

^b^*P*<.01 (2-tailed).

^c^PHQ-9: Patient Health Questionnaire-9 items.

^d^GAD-7: Generalized Anxiety Disorder-7 items.

^e^GMI: Gaming Motivation Inventory.

**Table 6:** Correlations between the Gaming Withdrawal Symptoms Questionnaire (GWSQ) and measures of discriminant validity.

Variable	GWSQ, *r* (95% CI)
SWLS^a^	0.28^b^ (0.09 to 0.45)
RYFF-18^c^	–0.36^b^ (–0.52 to –0.17)
GMI^d^ (Recreation)	–0.009 (–0.21 to 0.19)

^a^SWLS: Satisfaction With Life Scale.

^b^*P*<.01 (2-tailed).

^c^RYFF-18: Ryff Scales of Psychological Well-Being-18 items.

^d^GMI: Gaming Motivation Inventory.

As shown, the GWSQ demonstrated significant positive correlations with all measures. The strongest correlations were observed with the IGDS9-SF (*r*=0.476, 95% CI 0.321-0.606; *P*<.001) and the PHQ-9 (*r*=0.479, 95% CI 0.321-0.612; *P*<.001). The correlation with the GAD-7 was also significant, though somewhat weaker (*r*=0.415, 95% CI 0.246-0.560; *P*<.001). These correlations were precisely estimated, as evidenced by the consistently narrow 95% CIs, which increase confidence that these observed effect sizes accurately reflect the true population association. A similar pattern was found with the Introjected regulation subscale of the GMI (*r*=0.324, 95% CI 0.137-0.488; *P*<.001).

To assess the discriminant validity of the GWSQ, correlations were examined between the GWSQ and measures of constructs theoretically unrelated to gaming withdrawal symptoms. Specifically, correlations were calculated with the SWLS, the Ryff Psychological Well-Being Scale-18 items (RYFF-18), and the Recreation subscale of the GMI. Table 6 presents the Pearson correlation coefficients between the GWSQ and these measures.

Discriminant validity analysis demonstrated weaker, but still significant correlations between GWSQ and SWLS (*r*=0.28, 95% CI 0.09-0.45; *P*=.004), as well as a moderate negative correlation with RYFF-18 (*r*=–0.36, 95% CI –0.52 to –0.17; *P*<.001). Notably, there was no significant correlation between GWSQ and the GMI Recreation subscale (*r*=–0.009, 95% CI –0.21 to 0.19; *P*=.93), suggesting that the GWSQ does not capture general gaming motivation to play for recreational purposes.

Then, the reliability of all 3 subscales of the GWSQ was assessed. Table 7 presents Cronbach α, composite reliability (CR), and average variance extracted (AVE) statistics. The diagonal shows the square root of the AVE for each construct. The lower triangle of the last 3 columns of the table presents the correlation coefficients.

**Table 7:** Results of reliability analysis of Gaming Withdrawal Symptoms Questionnaire (GWSQ) and all subscales.

Subscale	CR^a^	Cronbach α^b^	AVE^c^	Motivational and Cognitive Withdrawal Symptoms, *r*	Affective Withdrawal Symptoms, *r*	Physical Withdrawal Symptoms, *r*
Motivational and Cognitive Withdrawal Symptoms	0.89	0.89	0.51	0.80	N/A^d^	N/A
Affective Withdrawal Symptoms	0.90	0.90	0.56	0.80^e^	0.96	N/A
Physical Withdrawal Symptoms	0.91	0.90	0.55	0.76^e^	0.75^e^	0.88
Total GWSQ	0.95	0.95	N/A	N/A	N/A	N/A

^a^CR: composite reliability.

^b^Desired coefficient value ≥0.75.

^c^AVE: average variance extracted (desired coefficient value ≥0.50).

^d^N/A: not applicable.

^e^*P*<.001.

The results of the reliability analysis and the explained variances for the total GWSQ and all scales indicate good internal consistency and validity. CR and Cronbach α values for each scale exceed the desired thresholds of 0.80, demonstrating strong reliability. Additionally, the AVE values were all>0.50, supporting construct validity. The square root of the AVE for each construct was compared with the correlation coefficients between the constructs. The square roots of the AVE values on the diagonal were greater than the corresponding interconstruct correlations in the lower triangle of the matrix, confirming the validity of the measurement.

#### Evaluation of Sten Standards and Interpretation Guidelines

To facilitate the interpretation of the scores obtained from the GWSQ, a standardization process was conducted. The raw scores were converted into sten scores to standardize individual performance relative to a reference group. To ensure accurate standardization, the reference group was selected to be representative of the general population, taking into account factors such as age, gender, and gaming experience. The raw scores were grouped into deciles, with sten scores ranging from 1 to 10, where lower scores (1-4) represent minimal withdrawal symptoms, average scores (5-6) indicate moderate withdrawal symptoms, and higher scores (7-10) suggest pronounced withdrawal symptoms [57]. The calculated stens for the GWSQ are presented in Table 8.

**Table 8:** Sten norms for the Gaming Withdrawal Symptoms Questionnaire and its subscales. Sten norms were derived from the large validation sample of 565 gamers (predominantly Polish-speaking participants). These norms are based on a general population of gamers rather than a clinical sample diagnosed with gaming disorder and should be interpreted accordingly when used in a clinical context. Sten scores are standard 10-point scores with a mean of 5.5 (SD 2).

Sten	Total score	Motivational Cognitive Withdrawal Symptoms	Affective Withdrawal Symptoms	Physical Withdrawal Symptoms
1	23-29	8-9	7-9	8-9
2	30-45	10-14	10-12	10-13
3	46-50	15-16	13-15	14-15
4	51-58	17-19	16-18	16-17
5	59-67	20-23	19-21	18-20
6	68-77	24-27	22-25	21-24
7	78-92	28-32	26-29	25-30
8	93-97	33-35	30-33	31-34
9	98-104	36-38	34	35-37
10	105-115	39-40	35	38-40

#### Interpretation Guidelines

- Low scores (sten 1-4): individuals scoring within this range exhibit minimal withdrawal symptoms related to gaming, suggesting that gaming cessation does not significantly impact their emotional, cognitive, or physical functioning.
- Average scores (sten 5-6): this range indicates moderate withdrawal symptoms. Individuals in this category may experience some discomfort when reducing or ceasing gaming, but these effects are typically not severe.
- High scores (sten 7-10): individuals in this range report pronounced withdrawal symptoms, including strong motivational urges to continue gaming, heightened emotional distress, and notable physical symptoms. These scores may indicate potential difficulties in regulating gaming behavior and warrant further psychological evaluation.

The same interpretive guidelines can be used to interpret scores on the 3 subscales (Motivational and Cognitive Withdrawal Symptoms, Affective Withdrawal Symptoms, and Physical Withdrawal Symptoms), providing a more qualitative assessment of results.

#### Results of GWSQ

Table 9 presents the descriptive statistics for the total score and the 3 subscales of the GWSQ. The dataset includes responses from 565 participants.

**Table 9:** Descriptive statistics of the Gaming Withdrawal Symptoms Questionnaire (GWSQ).

	n	Minimum-maximum	Mean (SD)
Motivational and Cognitive Withdrawal Symptoms	565	8-40	19.46 (6.85)
Affective Withdrawal Symptoms	565	7-35	19.56 (6.77)
Physical Withdrawal Symptoms	565	8-40	17.53 (6.55)
Total GWSQ score	565	23-115	56.57 (18.11)

The Motivational and Cognitive Withdrawal Symptoms subscale yielded scores ranging from 8 to 40 (mean 19.47, SD 6.86). The Affective Withdrawal Symptoms subscale had a similar range, with scores between 7 and 35 (mean 19.56, SD 6.78). The Physical Withdrawal Symptoms subscale exhibited scores ranging from 8 to 48 (mean 17.54, SD 6.55).

The overall GWSQ score varied between 23 and 115, with a mean score of 56.57 (SD 18.12). These results suggest variability in the severity of withdrawal symptoms among respondents.

## Discussion

### Principal Findings

This study aimed to develop and validate a comprehensive withdrawal inventory of GD, addressing the lack of standardized diagnostic criteria and measurement tools. We applied a multistage approach combining the development, validation, and normalization of the GWSQ through 2 studies.

Summarizing the contribution, we developed the GWSQ based on collected data from the scoping literature review [27]. Initially, the questionnaire consisted of 47 items encompassing the full spectrum of withdrawal symptoms (affective, cognitive, behavioral, and physical components). Psychometric validation, conducted in 2 separate empirical studies, identified a 3-factor structure of the GWSQ. The CFA results confirmed the extracted structure, which consists of the following subscales: Motivational and Cognitive Withdrawal Symptoms, Affective Withdrawal Symptoms, Physical Withdrawal Symptoms (*χ*^2^_227_=887.8; *P*<.001; comparative fit index=0.91; Tucker‐Lewis index=0.90; root-mean-square error of approximation=0.072, 90% CI 0.067-0.077; standardized root-mean-square residual=0.049). The results of the reliability analysis support the validity of the construct (Cronbach α=0.95; CR=0.95). Subsequently, the findings of the convergent and discriminant validity analysis confirm the relevance of the created tool. Therefore, the GWSQ is the first comprehensive and validated tool to measure withdrawal symptoms in GD.

The 3-factor structure of withdrawal symptoms identified in the GWSQ, comprising Affective Withdrawal Symptoms, Motivational and Cognitive Withdrawal Symptoms, and Physical Withdrawal Symptoms, aligns with existing conceptualizations of behavioral addictions and withdrawal theories. The multidimensional approach to withdrawal symptomatology in GD is consistent with the “components model of addiction,” where withdrawal is defined as the spectrum of psychological states and physiological effects [58,59]. These findings support the multidimensional nature of withdrawal presented in previous studies [27,28,35,37]. Furthermore, compared to prior research on withdrawal symptoms in GD, the GWSQ allows a more comprehensive identification of symptoms and their structure. Unlike the unidimensional approach, which primarily focuses on the affective component of withdrawal in existing IGD screening tools (eg, sadness and irritability; IGDS9-SF [29], 20-Item Internet Gaming Disorder Test [49], and Internet Addiction Test [30]), the GWSQ integrates affective, motivational, cognitive, and physiological components, broadening the understanding of withdrawal experiences. The expanded classification of symptoms in this study suggests that previous assessment tools may have provided an incomplete representation of the full spectrum of withdrawal experiences in GD.

This study contributes to the ongoing debate and criticism regarding the presence of withdrawal symptoms in behavioral addictions [13-16]. Compared to substance use disorders, withdrawal in GD has a more psychological nature, encompassing affective symptoms (eg, irritability, frustration, anger, and impatience), cognitive symptoms (eg, difficulty concentrating and preoccupation with gaming), and behavioral and motivational symptoms (eg, lack of motivation and inability to identify activities to do). Nevertheless, our research also points to the presence of physiological symptoms, including headaches, muscle pain, and sweating. However, the physiological symptoms observed in gaming withdrawal appear to be milder than those associated with substance withdrawal. This may be linked to the psychosomatic nature of emotions and their multidimensional structure, which includes cognitive, behavioral, and physiological components [60,61].

### Strengths of the Research and Its Implications

This study offers several methodological and conceptual advancements in the assessment of withdrawal symptoms in GD. One of its key strengths is the comprehensive approach to symptom identification, which was based on a systematic review of the literature. This ensured that the conceptualization of withdrawal in GD was grounded in empirical evidence and reflected the multidimensional nature of the phenomenon.

Regarding the methodological aspect of the research, although both surveys were conducted online, their strength lies in the direct individual contact established with respondents. Specifically, we recruited participants directly from gaming-related social media platforms such as Facebook, Reddit, Discord, and Steam Community, ensuring that those who participated were active gamers. This allowed for a more focused and engaged sample, minimizing the risks associated with the use of commercial panels, such as nontargeted or less motivated participants. Moreover, the high quality of the data collected is reflected in several aspects: the high reliability of the measurement tools, the consistency of results across the 2 surveys, and the indicators of high participant attention during the evaluation. Moreover, while most of the respondents were from Poland, the data included participants from various nationalities and geographic backgrounds.

Another notable strength is the rigorous validation process, which included both EFA and CFA conducted on independent samples. This 2-step approach ensured the robustness of the identified factor structure and provided strong psychometric evidence supporting the validity of the GWSQ. The CFA results confirmed an adequate fit of the 3-factor model, reinforcing the theoretical basis of the proposed classification. In addition, the study introduces norm-based scoring (sten scores), which enhances the practical applicability of the tool. By providing normative data, the GWSQ allows for a more precise interpretation of individual scores, facilitating both clinical and research applications. This is particularly valuable for distinguishing between different levels of withdrawal severity, aiding in the early identification of GD, as well as serving as a marker for more severe cases of the disorder [38].

Additionally, the high internal consistency (Cronbach α and CR) and satisfactory construct validity of the GWSQ further attest to the reliability and accuracy of the developed measure. The observed positive correlations with established instruments assessing related constructs (eg, IGDS9-SF, PHQ-9, and GAD-7) and weaker or nonsignificant correlations with theoretically unrelated constructs (eg, SWLS and RYFF-18) support the tool’s convergent and discriminant validity.

From a practical perspective, the GWSQ can be a valuable addition to existing GD screening tools, such as the IGDS9-SF [29], by enhancing epidemiological research, identifying risk factors, and tracking the progression of GD [38]. In clinical practice, it can help differentiate between problematic gaming and high engagement, enabling more accurate diagnoses and tailored intervention plans based on the severity of withdrawal symptoms [19-22]. Additionally, the GWSQ can monitor symptom reduction throughout treatment and assist in the early detection of individuals at risk of developing GD and cooccurring mental health conditions, such as depression and anxiety [19,39,40]. Moreover, as a potentially change-sensitive tool, the GWSQ holds immense promise as a patient-reported outcome measure in formal clinical trials for future GD therapeutics, serving as a standardized end point for evaluating treatment efficacy. By providing a standardized measure of withdrawal symptoms, the GWSQ can inform the development of targeted prevention and treatment strategies, ultimately improving intervention outcomes [62].

### Limitations and Future Directions

Despite its contributions, this study has certain limitations that should be considered when interpreting the findings and assessing their generalizability. First, the sample in both validation studies was predominantly Polish, which may limit the applicability of the results to other cultural and gaming populations. Although there were participants from other nations, their numbers were limited. Given that withdrawal experiences can be influenced by sociocultural factors which can impact social obligations, interpersonal gaming dynamics and habits, as well as norms surrounding digital leisure, emotional expression (particularly the emotional and physical expression of distress), power dynamics, and gaming motivations, this limitation is particularly important, as the English version of the GWSQ was administered to a predominantly nonnative English-speaking sample. Future studies should assess the psychometric properties of the GWSQ in diverse cultural contexts, especially among native English-speaking populations, to ensure its cross-cultural applicability and validity. Additionally, this study included a diverse gaming sample, with substantial variation in time spent on gaming and related activities. While this diversity increases ecological validity, it may also introduce interpretative challenges. Differences in gaming engagement levels could influence withdrawal symptom severity. Future research should consider more homogeneous subsamples or analyses by subgroup (eg, casual vs highly engaged players) to better capture the relationship between gaming behavior and withdrawal symptoms.

Moreover, the sten norms presented in Table 9 are based on the reference group that was representative of the general population by age, gender, and gaming habits. They provide a standardized interpretation of GWSQ scores, enabling comparisons between individuals. However, future work should focus on the applicability of these norms to clinical or high-risk groups, such as individuals with GD. Further studies are required to assess the applicability of these norms in clinical and treatment-seeking populations. Furthermore, replicating studies with more specific samples of players engaged in particular games or game mechanics could provide more concrete insights into their impact on withdrawal symptoms. Studies show that reward-based, competitive, and multiplayer genres, such as multiplayer online battle arena, first-person shooter, and massively multiplayer online role-playing game, are usually related to higher withdrawal symptoms than casual games [63,64]. Specific mechanics such as reward loops, ranking systems, “just one more turn/quest” behavior, unclear progression, or cliffhangers may trigger craving in situations of negative disengagement [65]. Future research should also investigate how specific withdrawal symptoms vary across different video game genres.

Subsequently, the study relied on self-report measures, which, although standard in psychometric research, are subject to recall bias and social-desirability effects [66]. Self-reported withdrawal symptoms may not fully capture the complexity of the phenomenon. As the study asked participants to reflect on a period in which they desired but were unable to play video games, it may not fully reflect the real-time dynamics of withdrawal. Future research could benefit from incorporating multimethod approaches and diverse study designs, including behavioral or physiological assessments and experimental methods, where participants attempt to withdraw from gaming under controlled conditions. This approach could provide more accurate insights into the withdrawal process. Furthermore, it should be acknowledged that IGDS9-SF, which was used as a primary validation tool, measures IGD—a related but distinct construct from GD. Therefore, correlations observed in the study with the GWSQ should be interpreted with this distinction in mind.

Building upon these considerations, additional directions for future research should aim to further explore the role of gaming withdrawal symptoms. First, longitudinal studies are needed to assess the temporal stability of withdrawal symptoms and the predictive validity of the GWSQ. The cross-sectional nature of this study does not allow conclusions about the persistence or fluctuation of symptoms over time. Examining how withdrawal symptoms evolve in relation to gaming habits, psychological states, and external influences will be essential for understanding their long-term impact. Additionally, future research should explore the underlying neurobiological and psychological mechanisms of gaming withdrawal. Neuroimaging studies, such as functional magnetic resonance imaging or electroencephalography, could provide insight into the brain activity associated with withdrawal symptoms, while neurophysiological assessments (eg, autonomic nervous system measures) could help differentiate the psychological and biological components of withdrawal. Experimental designs manipulating gaming access could help establish causal relationships. Finally, qualitative studies exploring the lived experiences of individuals undergoing withdrawal could reveal psychological processes and coping strategies not captured through standard questionnaires.

### Conclusions

This study introduces the first validated instrument specifically designed to assess withdrawal symptoms in GD, addressing limitations of existing measures that primarily focus on affective distress or general symptom severity. It provides empirical support for the multidimensional nature of gaming withdrawal by validating the GWSQ as a reliable and valid assessment tool. The findings highlight the importance of considering withdrawal as a core diagnostic feature of GD, demonstrating its affective, cognitive, motivational, and physiological components. The GWSQ’s strong psychometric properties, confirmed through EFA and CFA, reinforce its use for both research and clinical applications.

From a practical standpoint, the GWSQ offers a standardized method for identifying withdrawal symptoms, aiding early detection, diagnosis, and treatment monitoring. Its application extends to epidemiological research, thereby enabling a better understanding of GD risk factors and progression. Furthermore, the tool has the potential to differentiate problematic gaming from high engagement and support clinicians in tailoring therapeutic approaches. Future research should focus on cross-cultural validation, longitudinal assessment of withdrawal symptoms, and the integration of biopsychosocial correlates to further enhance the use of the GWSQ.

## Data Availability

In line with the best practices, the preregistration for the studies is available for download on the Open Science Framework [68]. The datasets generated or analyzed during this study are available from the corresponding author on reasonable request.

## Appendix

Multimedia Appendix 1 Questionnaire and STROBE checklist.

## Abbreviations

APA: American Psychological Association
AVE: average variance extracted
CFA: confirmatory factor analysis
COSMIN: Consensus-Based Standards for the Selection of Health Measurement Instruments
CR: composite reliability
DSM-5 TR: Diagnostic and Statistical Manual of Mental Disorders, Fifth Edition, Text Revision
DSM-5: Diagnostic and Statistical Manual of Mental Disorders, Fifth Edition
EFA: exploratory factor analysis
F1: motivational and cognitive factor
F2: affective factor
F3: physical factor
GAD-7: Generalized Anxiety Disorder-7 items
GD: gaming disorder
GIS: Gaming Involvement Scale
GMI: Gaming Motivation Inventory
GWSQ: Gaming Withdrawal Symptoms Questionnaire
ICD-11: International Classification of Diseases, Eleventh Edition
IGD: internet gaming disorder
IGDS9-SF: Internet Gaming Disorder Scale-Short Form
PHQ-9: Patient Health Questionnaire-9 items
RYFF-18: Ryff Psychological Well-Being Scale-18 items
STROBE: Strengthening the Reporting of Observational Studies in Epidemiology
SWLS: Satisfaction With Life Scale
